# Hypnosis in patients with perceived stress – a systematic review

**DOI:** 10.1186/s12906-017-1806-0

**Published:** 2017-06-19

**Authors:** S Fisch, B Brinkhaus, M Teut

**Affiliations:** 1 Institute for Social Medicine, Epidemiology, and Health Economics, Charité Universitätsmedizin Berlin, Luisenstr. 57, 10098 Berlin, Germany; 2 Psychotherapy Outpatient Clinic, Daruper Straße 14, D-48653 Coesfeld, Germany

**Keywords:** Hypnosis, Hypnotherapy, Stress, Perceived stress, Stress reduction, Systematic review

## Abstract

**Background:**

Although hypnosis and hypnotherapy have become more popular in recent years, the evidence for hypnosis to influence perceived stress is unclear. In this systematic review we searched and evaluated randomized clinical studies investigating the effect of hypnosis on perceived stress reduction and coping.

**Methods:**

The Cochrane Central Register of Controlled Trials, the Cochrane Database of Systematic Reviews, the Database of Abstracts of Review of Effects, EMBASE, Medline, PsycINFO, PSYNDEX and PubMed were systematically screened from their inception until December 2015 for randomized controlled trials (RCTs) reporting about hypnosis or hypnotherapy for stress reduction in healthy participants. Risk of Bias was assessed according the Cochrane Collaboration recommendations.

**Results:**

Nine RCTs with a total of 365 participants met the inclusion criteria and were included in this review. Most included participants were medical students, predominantly female (*n* = 211). Mean age of participants ranged in most studies between 20 and 25 years, in three studies the mean ages were between 30 and 42 years. Perceived stress was measured by a wide range of psychological questionnaires including Face Valid Stress Test, Stress Thermometer, and immunological data was collected. All nine included studies used explorative designs and showed a high risk of bias. Six out of nine studies reported significant positive effects of hypnosis for stress reduction in the main outcome parameter compared to control groups (3 active controls, 3 no therapy controls). Immunological outcomes were assessed in six studies, the results were inconclusive.

**Conclusions:**

Due to exploratory designs and high risk of bias, the effectiveness of hypnosis or hypnotherapy in stress reduction remains still unclear. More high quality clinical research is urgently needed.

## Background

Psychological distress and stress-related diseases are considered to be an important health issue world wide [1, 2] (Global Organization for Stress). Selye – one of the pioneers of stress research – postulated that “there is an integrated syndrome of closely interrelated adaptive reactions to non-specific stress itself; this has been termed the ‘General Adaption Syndrome’. It develops in three stages: the ‘Alarm Reaction’, the Stage of Resistance, and the Stage of Exhaustion. In the biological sense stress it is the interaction between damage and defense, just as in physics tension or pressure represents the interplay between a force and the resistance offered to it” [3]. In the 1950s and 1960s Lazarus developed a more cognitive model of stress with more focus on the meaning of appraisal of the stressors by the individual [4]. Heinrichs, Stächele, and Domes provide a modern and more operational definition of “stress” which includes important stress theories and models (e.g. by Selye as well as Lazarus) and their applicability in the clinical context: “Stress results from a threat of physiological and / or psychological integrity of a person, which causes an adaptive physiological, behavioral, emotional, and cognitive response. The individual amount of stress response is determined by integrating the individual psychobiological stress reactivity, the subjective threat assessment and the assessment of available coping resources. Stress thus represents a short-term imbalance between perceived burdensome requirements and regulation of available resources. Chronic stress occurs when the adaptive reaction does not lead to cope with the stressor and the imbalance remains.” [1] The stress associated symptomatology may include physiological (increased heart rate, muscular tension), cognitive (brooding, difficult concentrating), emotional (anxiety, anger, touchiness, lability) and social symptoms (social withdrawal).

Stress management techniques today play an important role in clinical work. In recent years modern clinical hypnosis and hypnotherapy have become increasingly popular and received greater attention worldwide. Hypnosis is defined as “a state of consciousness involving focused attention and reduced peripheral awareness characterized by an enhanced capacity for response to suggestion” [5]. Hypnotherapy is defined as “the use of hypnosis in the treatment of a medical or psychological disorder or concern” [5] and additionally includes therapeutic conversation using hypnosystemic language and a resource-activating and solution-oriented attitude. Hypnotizability is defined “an individual’s ability to experience suggested alterations in physiology, sensations, emotions, thoughts, or behavior during hypnosis.” [5].

Cognitive-behavioral methods and also mindfulness-based stress reduction methods have been thoroughly investigated for their effectiveness and benefits for stress reduction [6–8]; several evidence-based cognitive-behavioral stress management trainings are available [9–15]. In comparison with those approaches the clinical effectiveness of hypnotherapeutic methods for stress reduction is still quite poorly investigated, although there is an abundance of practical literature from the psychotherapeutic practice to teach hypnotherapy interventions for coping with stress [16–19].

The aim of this systematic review is to investigate the actual status of clinical research on hypnotherapeutic approaches for stress reduction especially which psychotherapeutic interventions were used, which outcomes were assessed, how the effectiveness was measured and what effects were observed.

## Methods

PRISMA guidelines for systematic reviews and meta-analysis [20] and the recommendations of the Cochrane Collaboration were followed [21] for this systematic review.

### Eligibility criteria for studies to be included

Studies had to meet the following criteria to be eligible for the review:

1. Studies: Randomized controlled trials (RCTs) were included
2. Participants: Adult healthy participants (aged >18 years) with elevated perceived stress levels at present or future (preventive and therapeutic)
3. Interventions: Comparison of hypnosis/hypnotherapy with another active intervention or a no therapy group
4. Outcomes: Questionnaires measuring perceived stress, in addition immunological parameters possible

### Search methods

The Cochrane Central Register of Controlled Trials, the Cochrane Database of Systematic Reviews, the Database of Abstracts of Review of Effects, EMBASE, Medline, PsycINFO, PSYNDEX and PubMed were searched from their inception (Medline from 1946, EMBASE from 1947, PsycINFO from 1966 and PSYNDEX from 1978) until December 2015 without language restrictions. The key words for our search were: “hypnosis” OR “hypnotherapy” AND “stress management” AND “study”, “hypnosis” OR “hypnotherapy” AND “stress reduction” AND “study”. Additional searches were carried out in April 2017. In PubMed we also did the search with the terms “hypnosis” OR “hypnotherapy” AND “stress” AND “trial”. The Cochrane Central Register of Controlled Trials and the Cochrane Database of Systematic Reviews were furthermore searched with “hypnosis” OR “hypnotherapy” AND “stress”. Additionally, the reference lists of identified original and review articles were searched manually. Abstracts of identified records were screened, and the complete articles of potentially eligible studies were carefully screened by two investigators (SF, MT) independently to determine whether they met the eligibility criteria. Discrepancies were discussed until consensus was reached.

### Data extraction and management

Data on included patients, design, interventions and controls, outcomes and results were extracted by SF using a predefined data extraction form. The results were reviewed by a second investigator (MT), discrepancies were discussed until consensus was reached. Study authors were contacted for additional information if necessary.

### Assessment of risk of bias

Included RCTs were assessed for risk of bias according the Cochrane collaboration’s tool for assessing bias [21]. This included the domains sequence generation, blinding of participants and personnel, blinding of outcome assessment, incomplete outcome data, selective reporting, and other sources of bias. Risk of bias was assessed for each domain as low, unclear and high risk of bias (SF). The assessments were reviewed by a second investigator (MT) and discrepancies were discussed until a consensus was reached.

## Results

### Literature search

**Fig. 1:**
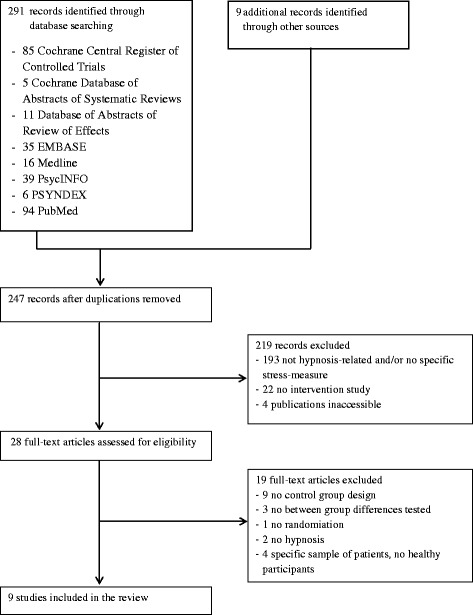
Flowchart of the results of the literature research

The search process is presented in the flow chart (Fig. 1). We identified 247 abstracts of studies after removing duplicates. 219 records were excluded: 193 were not hypnosis-related and/or used no specific stress-measure and/or there were no healthy participants, 22 were no trials and 4 publications were inaccessible. 28 full-text articles were assessed for eligibility. After investigating full text manuscripts 19 papers had to be excluded due to methodological limitations: nine studies without control group design [22–30], three without reporting between group differences of testing [31–33]. In one study there was no randomization of subjects to groups [34]. In two studies the study intervention was not hypnosis, but a kind of autogenic training [35] and Reiki in combination with positive imagery respectively [36]. In four studies there were no healthy participants included, but samples of patients with specific disease conditions [37–40]. The remaining nine studies were included in this systematic review [41–49].

### Study characteristics

Characteristics of the included studies with samples, designs, interventions, type of control conditions, outcome measures, results, and information about the study quality are shown in Table 1.

**Table 1:** Characteristics of studies included

Authors, Year	Sample, Sample Size, Gender, Mean Age (SD, range)	Design, Number of Groups, Sample points	Type of Intervention	Type of Control Condition	Outcome Measures	Results (Between group differences)	Study Quality (Strengths, Limitations)
Barling, NR and Raine, SJ (2005) [41]	60 healthy volunteers (14 males, 46 females) Mean age 41.5 (range 17–63) No information about further demographic data in the sample	RCT Three experimental groups, one control group Two sample points: • pre-test before the intervention • post-test after three weeks of self-practice	1) PMR 2) PMR + guided imagery (GI) 3) PMR + GI + deep trance (DT) with specific mind-body healing language Tape with recorded intervention for self-practice	No intervention	Burnout Assessment Test (BAT) [60] Depression Anxiety Stress Scales (DASS) [61] Saliva Immunoglobulin A (sIGA) by “sucking in their mouth”	• Significant condition effect for change in burnout (F(3, 14) = 8.46, *p* > 0.001), depression (F(3, 14) = 5.13, *p* > 0.003), anxiety (F(3, 14) = 6.62, *p* > 0.001) and stress (F(3, 14) = 4.02, *p* > 0.01); • Significant differences in changes in burnout, anxiety, and depression between the control group and the DT group (no p-score reported)	+ One-way analysis of variance (ANOVA) Multiple regression analyses - Small sample size No information about further demographic data and health-related behaviors in the sample No information about way of random assignment to the experimental groups No information about results of post hoc-comparisons between groups
Cardena et al. (2013) [42]	56 healthy volunteers 10 males, 46 females Mean Age 31.13 (SD = 10.00; range 20–61) 44 students, 11 worked, 1 unemployed 21 participants dropped out	RCT One experimental group One control group Three times of measurement: 1. before the hypnotic intervention 2. after two weeks after the hypnotic intervention for group 1 3. after further two weeks after the hypnotic intervention for group 2	Hypnotic intervention: participants were asked to listen to a recording (length 23 min) at least once a day for 14 days. The tape script consists of an general induction with a focus on breathing, counting a deepening procedure; imagining of specific place of security, peace, and control; creating a personalized “anchor”, posthypnotic suggestions	Waiting list control group	Perceived Stress Scale (PSS) [53] Shirom-Melamed Burnout Questionnaire (SMBQ) [62] Ways of Coping Questionnaire (WCQ) [63] General Health Questionnaire 12 (GHQ-12) [64]	• Less stress (t(33) = -2.751, *p* < .01) and better overall health (t(22.5) = -3.159, *p* < .004) in hypnosis than in control group	+ precise information about exclusion criteria and way of randomization ANOVAs - Many drop-outs
Gruzelier et al. (2001) [43]	28 volunteer pre-clinical medical students 17 males, 11 females Mean age 20.1	RCT one experimental group one control group Two sample points • Baseline: four weeks before examination • Exam: during the exam period	Group hypnosis three weeks before examinations Tape with recorded intervention for self-practice Hypnotic induction: visual fixation, relaxation and deepening exercises, instructions aimed at improving immune function and to mobilise resources by increasing alertness, energy and concentration (length 20 min)	No intervention	Lymphocyte counts (CD3, CD4, CD8, CD 8/4%, CD 19, NKC) Cortisol Life style questionnaire Emotional state: scales of tension, calmness, energy and tiredness) [65] State anxiety scale [66] Activated and withdrawn personality scales [67]	• Immunological measures: reduction in NK cells with exam stress in controls, non-significant increase with hypnosis (F(2, 25) = 6.03, *p* < .007); • Mood: no group differences in mood changes (no F- and p-scores reported); • ratings of energy higher at exam time in hypnosis than in control group (F(1, 26) = 6.16, *p* < .01)	- Small sample size Some results not reaching significance are reported and interpreted as positive results
Kiecolt-Glaser JK et al. (1986) [44]	34 first-year medical students 22 male, 12 female Mean age 23.5	RCT One experimental group One control group Two sample points: 1. one month before examination 2. on the day of examinations	Hypnotic/relaxation group during lunch hours; 5–10 sessions in 2.5 weeks before the second blood draw First hypnotic session: overview of hypnosis, group induction; Each subsequent session: series of deepening exercises used in the initial session, self-hypnosis, progressive relaxation, autogenic training, various imagery exercises, suggestions for greater relaxation throughout the day and enhanced comprehension and retention of academic material (Sessions lasted 25–40 min); Written manual that specified the content and order of components within each the sessions and request for daily practice	No intervention	Brief Symptom Inventory (BSI) [54]: changes in global stress State Loneliness Scale [68] Assessment of the degree of relaxation achieved during each session (self-rating 1–9) Academic performance Immunological data Percentage of helper/inducer cells Percentage of suppressor/cytotoxic cells Helper/suppressor-cell ratio NK activity Nutritional data: Albumin, TIBC, Transferrin	• BSI: significant increases in anxiety (*p* < .01), obsessive-compulsive symptomatology (*p* < .05) and in the global severity index (*p* < .05) only in no-intervention group No main effect for group and no group x time interaction in • Loneliness • Changes in health-related behaviors • Academic performance • Immunological data • Nutritional data	+ Repeated-measures analyses of variance design multiple regression analyses - Small sample size
Kiecolt-Glaser JK et al. (2001) [45]	34 students who obtained a score of 7 or higher on both scales (HGSHS-A and SHSS-C) among 130 students volunteered for the initial screening session 14 male, 19 female Mean age 23.48 (SD +/− 1.97)	RCT One experimental group One control group Two sample points: 1. within the first few days of the quarter 2. three days before the first major academic examination of the term	5–10 sessions during lunch hours that began 8 days before the second blood draw First hypnotic session: overview of hypnosis, group induction; Each subsequent session: series of deepening exercises used in the initial session, various imagery exercises, suggestions for greater relaxation throughout the day and enhanced comprehension and retention of academic material (sessions lasted 25–40 min); Written manual that specified the content and order of components within each the sessions and request for daily practice	No intervention	Self-rating of anxiety (0–10) prior to each of the group inductions Current relaxation after completion of group induction Perceived Stress Scale [53] Positive and negative Affect Schedule PANAS [69] New York University Loneliness Scale [70] Immunological Assays Blastogenic response to phytohemagglutinin (PHA); blastogenic response to concanavalin A (Con A); T-lymphocytes, NK cells, macrophage/monocytes, IL-1β	No significant group x time interaction, no group effect for • Stress (PSS) and negative affect (PANAS) • Loneliness • Immunological data: stable values for PHA stimulation (F(1, 31) = 4.94, *p* < .04), Con A concentrations (F(1, 31) = 4.26, *p* < .05), for CD3 + T-lymphocytes (F(1,31) = 5.76, *p* < .03)- and CD4+ T-lymphocytes (F(1, 31) = 6.05, *p* < .03) in hypnosis and declines for control group; no significant group effects or group x time interaction for CD8 + −, NK-cells and IL-1β	- Small sample size
Naito A et al. (2003) [46]	48 students (39/48 medical students); 22 males, 26 females; Age range 19–23 years with one participant of 37 years Participants were paid £30 at the end of the study.	Prospective randomized controlled trial Three groups • Stress reduction training with self-hypnosis • Johrei • Mock neurofeedback relaxation control Two sample points: • Baseline: before training • Exam: 1–2 months later as exams approached	4 Weekly sessions during a 1-month intervention period Self-hypnosis training: subjects learnt a Spiegel-type eye-roll for instant relaxation first and then a slower relaxation-type induction; subjects were taught a basic immune imagery, and two anxiety management techniques; standard tape-recording using a relaxation induction and imagery description and request for self-hypnosis three times a day Johrei healing method: introduction to Johrei philosophy and the coreprinciples needed such as healing oneself by healing others; the subjects were requested to practice Johrei daily with a partner; the practitioner imagines light entering his body being concentrated through his hands towards the recipient and moves his hands slowly from head down to kidney area without touching the recipient	8 mock neurofeedback sessions over 1 month	Self-reported stress [53] Personalised Emotional Index: practice and mood data Peripheral blood lymphocytes: CD4+ T cells, CD8+ T cells, CD56+ Natural Killer cell percentages (NK cells) and NK cell cytotoxic activity	Natural Killer cells: increase only in Johrei, no change in hypnosis and relaxation (F(1,33) = 5.86, *p* = .007) CD8+ T cells: the extent of increase significant greater in hypnosis than in relaxation, but not than in Johrei (F(1,33) = 3.02, *p* = .063). CD4+ T cells: decline only in Johrei, no change in hypnosis and relaxation (F(2, 32) = 4.71, *p* = .016)	+ Repeated-measures ANOVA followed by paired comparisons with non-parametric tests - Small sample size No information about health-related behaviors in the sample Due to very small samples it is impossible to draw reliable conclusions of results of ANOVA with three factors
Stanton HE (1989) [47]	40 high school teachers No information about further demographic data in the sample	Prospective randomized controlled trial One experimental group One control group Three sample points: • Before treatment • Immediately after treatment • 12 months after treatment	4 weekly sessions involving a hypnotic induction and 10 positive suggestions derived from key elements of Rational-Emotive Therapy based on a reformulation of Ellis’s challenges to his clients’ irrational ideas (Ellis and Grieger, 1977) • 1. Session (1 h): hypnotic training and introduction to the 10 suggestions • 2.-4. Session (0.5 h): standardized induction (breath, counting, body relaxation, pleasant scene imagery) and five repetitions of the suggestions	4 weekly sessions with discussingstress reduction methods	Face Valid Stress Test Level of reasonable thinking: Teacher Idea Inventory [71]	• Teacher Idea Inventory: significant less irrational thinking in hypnosis than in control group after treatment (F(1, 19) = 32.61, *p* < .01) and at 12-month follow-up (F(1, 19) = 30.65, *p* < .01). • Face Valid Stress Test: significant lower stress level in hypnosis than in control group at 12-month follow-up (t(19) = 5.08, *p* < .001)	+ Repeated-measures ANOVA followed by paired comparisons with non-parametric tests -
Stanton HE (1991) [48]	30 secretaries from a large business firm Age range 27–43	RCT One experimental one control group Three sample points: • Before treatment • Immediately after treatment • 2 months after treatment	Two sessions (1. 50 min, 2. 25 min) while participants listened to an standardized tape which guided them through five stress-reduction steps: • physical relaxations induced by concentration upon the breath • mental calmness induced by imagining the mind as a pond into which one can drop concepts such as calmness, confidence as stones • disposing of “rubbish” as fears, doubts, and worries down a chute • removal of a barrier of self-destructive thoughts, fears of failure • enjoyment of a special place and remake the day	Two sessions of the same duration discussing stress management procedures (stage 1) After the 2 months follow-up of the experimental group the control group experienced the same two treatment sessions (stage 2)	Stress thermometer [72] Anecdotal reports	• Significantly greater stress reduction in hypnosis than in control group immediately after the treatment (Scheffé F(14) = 3.64, *p* < .01) and at 2-month follow-up (Scheffé F(14) = 3.47, *p* < .01)	+ Repeated measures ANOVA - Small sample size No standardized, validated self-report measures
Whitehouse et al. (1996) [49]	35 first-year medical students 14 male, 21 female Mean age 24.8	Prospective randomized controlled trial One experimental group One control group Four sampling points: • Orientation • Late semester • Exam stressor, • Recovery	19-week investigation Self-hypnosis training condition (*n* = 21) 14 sessions à 90 min around the noon hour, one day per week request for self-hypnosis exercises at least 15 min each day.	No treatment Subjects filled out the same daily diaries	Psychosocial data Profile of Mood States [73] Brief Symptom Inventory (BSI) [54] UCLA Loneliness Scale [74] Immunologic data • T, B, monocyte, granulocyte, NK, T4, T8, helper-inducer, and suppressor-inducer cells • Mitogen-induced lymphocyte stimulation by ConA, PHA and PWM	• BSI: significant less anxiety to exam period in hypnosis than in control group (F(3, 96) = 2.96, *p* < .05) No between group differences • UCLA loneliness scale • Immunologic data	+ Immunological data: repeated-measures multivariate analyses of variance (MANOVAs) Psychosocial data: univariate repeated-measures ANOVAs

### Setting and participant characteristics

The nine RCTs with a total of 365 participants included in this review were conducted in Australia [41, 47, 48], in the USA [44, 45, 49], the United Kingdom [43, 46] and Sweden [42].

Most participants were medical students [43–46, 49]. In the study reported by Cardena et al. 79% of the sample were students; another 20% were regular employed persons [42]. Barling and Raine recruited participants with the help of poster announcements around local fitness and health centres and did not provide further sociodemographic data of their participants [41]. One study included high school teachers [47], another included secretaries from a large company [48].

More participants were females (*n* = 211); two studies had predominantly male participants [43, 44]; one study did not report on the gender of participants [47]. Mean age ranged in most studies between 20 and 25 years, in three studies the mean ages were between 30 and 42 years [41, 42, 48], one study did not report on the age of participants [47].

Six RCTs used no intervention control groups (including waiting-list group design) [41–45, 49], two studies compared hypnosis with active control interventions, e.g. interventions stress reduction education [47, 48] and mock neurofeedback sessions [46]. Two studies used a three- and four-armed-design respectively (Naito: 1. hypnosis, 2. Johrei (a Japanese visualization and healing technique), 3. neurofeedback [46]; Barling: 1. PMR, 2. PMR + guided imagery (GI), 3. PMR + GI + deep trance, 4. no intervention) [41].

Six studies assessed the hypnotizability and susceptibility of participants, respectively at baseline and assessed the effect of this variable on outcome measures: The measure most frequently used in the trials was the Harvard Group Scale of Hypnotic Susceptibility, Form A, by Shor and Orne [42, 43, 45, 46, 49, 50]. In contrast, Barling and Raine used the Stanford Hypnotic Clinical Scale for Adults by Morgan and Hilgard [41, 51]. Kiecolt-Glaser et al. who wanted to examine especially high susceptible participants, used the Harvard Group Scale of Hypnotic Susceptibility, Form A, by Shor and Orne [50] and as a second measure the Stanford Hypnotic Susceptibility Scale, Form C by Weitzenhoffer and Hilgard [45, 52].

### Limitations/risk of bias

All included studies used exploratory designs and, following the publications, showed a high risk of bias (see Table 2) according to the Cochrane collaboration’s tool for assessing bias (compare table 2) [21]. All studies were reported as RCTs, but details of randomization sequence generation and allocation concealment were only reported by Cardena et al. [42]. No study reported blinding procedures of patients or therapists or blinding of outcome assessments. Only two studies reported on a low drop out rate [41, 44], the other studies had an unclear or high risk of attrition bias. However, the risk of outcome reporting bias was low in most studies. In the studies by Barling and Raine and Gruzelier et al. selective reporting due to incomplete presentation of results is possible with a high risk of outcome reporting bias [41, 43].

**Table 2:** Risk of bias

	Random sequence generation (Selection Bias)	Allocation concealment (Selection Bias)	Blinding of participants and personnel (Performance Bias)	Blinding of outcome assessment (Detection Bias)	Incomplete outcome data (Attrition Bias)	Selective reporting (Reporting Bias)
Barling, NR and Raine, SJ (2005) [41]	?	?	−	−	+	−
Cardena et al. (2013) [42]	+	−	−	−	−	+
Gruzelier et al. (2001) [43]	?	−	−	−	?	−
Kiecolt-Glaser JK et al. (1986) [44]	?	−	−	−	+	+
Kiecolt-Glaser JK et al. (2001) [45]	?	−	−	−	−	+
Naito A et al. (2003) [46]	?	−	−	−	−	+
Stanton HE (1989) [47]	?	−	−	−	?	+
Stanton HE (1991) [48]	?	−	−	−	?	+
Whitehouse et al. (1996) [49]	?	−	−	−	?	+

Key: + low risk of bias, − high risk of bias,? unclear risk of bias

Most included studies had very small samples sizes. Due to the exploratory nature of the trials, none of the studies applied and reported sample size calculations.

### Interventions

Three studies used a combination of one or more sessions of group-hypnosis in combination with the use of an audiotape of the recorded interventions, that participants were instructed to use for home-based self-hypnosis for several weeks. The advised frequency of self-practice hypnosis varied in the trials from no specifications [41], to “at least 3 times a week” [43] to “3 times a day” [46]. In one study only an audiotape with hypnosis intervention was given to the participants for self-practicing at least once a day for 14 days [42]. Three studies used a combination of 5–10 and 14 sessions of group-hypnosis respectively and requested participants to practice self-hypnosis daily without an audiotape [44, 45, 49]. Kiecolt-Glaser et al. gave their participants a written manual as guide book to self-hypnosis. In two of the studies 2 and 4 group sessions were conducted, respectively, without further self-hypnosis training [47, 48].

Most studies used a hypnosis protocol consisting of the following procedures: Hypnotic induction, deepening exercises, imagery exercises, posthypnotic suggestions. The hypnotic induction techniques included focusing the attention on breathing [42, 47, 48], visual fixation [43], and the Spiegel-type eye-roll [46]. Kiecolt-Glaser et al. did not report on their induction techniques [44, 45].

For deepening trance several techniques were used; Cardena et al. and Stanton used counting [42, 47]. Some studies used imagery of places of security, peace, or control [42], others did not specify the kind of imagination used [41, 44, 45]. Naito et al. combined their relaxation induction with a specific guided imagery of the immune system which was not further described [46]. Stanton developed a hypnosis procedure using several creative imagery interventions to aim at physical relaxation, mental calmness, disposing of fears and doubts, removal of self-destructive thoughts, and remaking the day [48] (for further details see Table 1).

Several studies used posthypnotic suggestions aimed to improve immune function [41, 42] and to activate resources by increasing alertness, energy and concentration [42], greater relaxation throughout the day and enhanced comprehension and retention of learning content [44, 45]. Stanton applied a combination of hypnosis and Rational Emotive Therapy (RET) and gave his participants ten positive suggestions derived from key elements of RET which were based on a reformulation of Ellis’ challenges to his clients irrational ideas [47].

Barling and Raine reported that they compared three types of interventions, but did not explain in detail how their interventions were structured. They used Progressive muscle relaxation (PMR) vs. PMR and guided imagery vs. PMR and guided imagery and “deep trance with mind-body healing language” [41]. Whitehouse et al. did not report the type of interventions they used [49].

### Outcome measures

Stress was measured and operationalized by a wide range of psychological questionnaires [41–45, 49] (compare Table 1), Face Valid Stress Test and Stress Thermometer, respectively [47, 48], and immunologic data [41, 43–46, 49].

Two studies used the Perceived Stress Scale (PSS) by Cohen et al. [53] to assess stress [42, 45], two studies used the Brief Symptom Inventory (BSI) by Derogatis and Spencer [54] to assess global stress [44, 49]. The remaining studies used different questionnaires or self-ratings for assessing burnout, depression, anxiety, positive and negative affects, and/or mood states as measurements of stress.

### Study results

#### Psychological outcomes

Barling and Raine reported significant differences in changes of burnout, anxiety and depression between hypnosis and control group [41]. Cardena et al. reported less stress and better overall health in the hypnosis group compared to control group [42].

Kiecolt-Glaser et al. described in students a significant group x time interaction with non-significant changes between baseline and examination period within the relaxation group compared to significant increases in anxiety, obsessive compulsive symptomatology, and in the global severity index of Brief Symptom Inventory by Derogatis and Spencer [54] in the no-intervention group during examination period [44].

Whitehouse described a significant group x time interaction with significant lower results in the anxiety scale of Brief Symptom Inventory by Derogatis and Spencer [54] in the self-hypnosis group compared to the waiting list control group in students during examination period [49].

In the study by Stanton teachers in the hypnosis group experienced a significant reduction in irrational thinking compared to control group (4 sessions discussing stress reduction methods) directly after treatment, but also at a 12-month-follow-up, where the intervention group had a significant lower stress level than the control group [47].

Stanton reported that secretaries in the hypnosis group had significantly greater stress reduction compared to control group (2 sessions discussing stress management procedures) immediately after the intervention and at the 2-month-follow-up [48].

No differences in clinical parameters between hypnosis and control were found in the studies by Gruzelier et al., and Kiecolt-Glaser et al. [43, 45].

#### Immunological outcome measures

Gruzelier et al. described a significant interaction between group and session with a reduction in NK cells with exam stress in controls (no intervention) compared to a non-significant increase with hypnosis [43]. Kiecolt-Glaser reported stable values of PHA stimulation (blastogenic response to phytohemagglutinin), Con A concentrations (blastogenic response to concanavalin A), CD3 + − and CD4 + T-lymphocytes for hypnotic-relaxation participants compared with declines for control group, for other immunologic parameters no significant group differences were observed [45].

Naito et al. reported that the extent of change of CD56+ NK cells and CD4+ T cells was significant greater in the Johrei group compared to the biofeedback group and to the hypnosis group [46].

No significant group differences in immunological outcomes were observed by Barling and Raine; Kiecolt-Glaser et al. and Whitehouse et al. [41, 44, 49].

#### Effects of hypnotizability/ susceptibility

Those studies, which investigates the effect of hypnotizability and susceptibilityon changes of psychological or immunological outcomes, respectively did not find any [41, 43, 45, 46, 49] or not more than scattered positive correlations [42] between hypnotizability measures and stress measures.

## Discussion

### Principal findings

To date only very few studies have investigated the effectiveness of hypnosis on stress reduction. Summarizing our findings, we found unclear evidence for the effectiveness of hypnosis in stress reduction in healthy subjects. This result is mainly due to methodological limitations of the available included studies, such as the use of exploratory designs, small sample sizes and incomplete reporting. However, six out of nine studies reported a significant reduction of perceived stress with hypnosis [41, 42, 44, 47–49], in three studies immunological changes were reported, yet due to different outcomes measured a conclusion is difficult to draw [43, 45, 46]. Altogether the included studies did not find significant correlations between the hypnotizability or susceptibility of participants and the amount of stress reduction. The one study that included only highly susceptible participants found no evidence of an effect of hypnosis on psychological measures of stress reduction at all [45].

### Strengths and limitations of this review

To our knowledge this is the first available systematic review on hypnosis for stress reduction in healthy subjects. It included only RCTs and only study designs with between group comparisons and included only studies clearly using hypnosis as intervention and using psychological outcome measures to assess perceived stress levels. Therefore we did not include studies on autogenic training which is described by some authors as a special and very standardized form of hypnosis [24, 55]. There may be more RCTs available for evaluation if the search would be extended to intervention strategies derived or inspired by hypnosis such as autogenic training or active imagination. We did also exclude research publications with diseased subjects and stress reduction as this was not the focus of our research questions.

### Methodological concerns of the included studies

All identified studies used exploratory designs and included small samples of participants. Therefore the external validity of the results is questionable. In fact, most of the subjects included in the studies were students. This could be seen as a potential source of community bias. It is unclear if the results of the studies can be generalized to the population. However, the results of this review are partly in line with previous reviews on stress-management-programs for medical students [56, 57] and for mental health nurses, respectively [58], that included several studies with different methods of stress management (i.a. hypnosis). They found evidence for the effectiveness of stress management programs for these samples, but criticized similar methodological concerns, e.g. “a lack of consistency of outcome measures across studies” [57] and “a lack of careful control in most studies, few validated outcome measures, and heterogeneous interventions” [56].

Most of the included exploratory studies investigated a high variety of outcome measures without defining primary outcomes in advance. Several studies conducted numerous post hoc analyses as a way of exploring the data [43–46, 49] with some positive and some negative results. In such cases, it is still very difficult to draw conclusions. Also a variety of different psychological outcome measures were used resulting in difficulties in comparison.

The fact that no study reported blinding procedures of patients or therapists or blinding of outcome assessments has to do with the nature of investigated treatment technique. People usually recognize if they are hypnotized, if direct and classical methods of hypnosis are used. An effort to create a form of control condition, that might appear to be hypnosis yet is not, is unrealistic to implement.

Our review clearly shows that research efforts about the effect of hypnosis on stress reduction in healthy subjects have never proceeded from the initial exploratory phase to a stage of confirmatory clinical studies.

One of our research questions was to find out which hypnotherapeutic interventions had been used successfully in the past. In summary the hypnotherapeutic techniques used as interventions in the included studies were very heterogeneous and were often not very well described. Most of the study interventions used aimed to increase relaxation. Some of them had their focus more on investigating the effect of hypnosis on the immune system rather than stress reduction [43–46]. Barling and Raine as well as Whitehouse et al. did not report in detail what kind of hypnotherapeutic techniques were used [41, 49]. Cardena as well as Gruzelier et al. only used basic hypnotherapeutic strategies such as imagining a place of peace and quiet in combination with some suggestions for improved immune functions [42, 43]. Some interventions aimed to specifically increase stress coping by improving the ability to relax physically and calm down mentally using various specific imagery exercises and suggestions [44–46]. But as Yapko stated, relaxation is “simply a stepping stone in the direction of facilitating more complex hypnotic experiences. … No one would simply do a relaxation process and then expect the client to undergo a painless surgery.” [59].

So at least in theory (and teaching) hypnosis offers specific possibilities and suggestions to improve one’s ability to cope with stress that go beyond simple relaxation techniques including many cognitive and behavioral components. Examples for those specific hypnotic interventions can be found at Stanton (1991), who used techniques aiming to improve one’s ability to protect oneself from stressors [48] or changing irrational cognitions about one’s performance orientation [47]. One of our hypotheses, derived from hypnotherapeutic theory, was that the effect of the interventions can be increased by the use of more specific and individualized suggestions. Our data is insufficient to discuss this hypothesis, but for future prospective trials it would be interesting to include arms with general relaxation techniques only and others with augmented specific suggestions and techniques.

### Implications for further research

Overall the role of hypnosis in stress reduction has to be investigated more thoroughly by using accurate research methods. For future trials we would recommend including the following aspects:

- Implementation of qualitative research in order to find out which hypnotherapeutic interventions are really used by psychotherapists in their daily clinical work and which outcomes are reported by patients.
- Development of standardized or semi-standardized (allowing individualization) hypnotherapeutic interventions together with stakeholders (e.g. therapists, patients). The use of well suited outcome measures for perceived stress, quality of life and other measures such as self-efficacy and others.
- A pre-testing of interventions and outcome measures using mixed methods research.
- The use of a confirmatory RCT design comparing active and semi-standardized hypnotherapeutic interventions with active control groups (e.g. behavioral therapy, empathic listening, listening to music) or no intervention control (e.g. waiting list). This may include group or individual interventions.

## Conclusion

Due to the exploratory nature and low quality of the included studies the effectiveness of hypnosis or hypnotherapy for stress reduction remains unclear. More high quality clinical research is needed.
